# Prediction of protein self-interactions using stacked long short-term memory from protein sequences information

**DOI:** 10.1186/s12918-018-0647-x

**Published:** 2018-12-21

**Authors:** Yan-Bin Wang, Zhu-Hong You, Xiao Li, Tong-Hai Jiang, Li Cheng, Zhan-Heng Chen

**Affiliations:** 1 Xinjiang Technical Institute of Physics and Chemistry, Chinese Academy of Science, Urumqi, 830011 China; 2 University of Chinese Academy of Sciences, Beijing, 100049 China

**Keywords:** Self-interacting proteins, Stacked long short-term memory, Deep learning, Dropout

## Abstract

**Background:**

Self-interacting Proteins (SIPs) plays a critical role in a series of life function in most living cells. Researches on SIPs are important part of molecular biology. Although numerous SIPs data be provided, traditional experimental methods are labor-intensive, time-consuming and costly and can only yield limited results in real-world needs. Hence,it’s urgent to develop an efficient computational SIPs prediction method to fill the gap. Deep learning technologies have proven to produce subversive performance improvements in many areas, but the effectiveness of deep learning methods for SIPs prediction has not been verified.

**Results:**

We developed a deep learning model for predicting SIPs by constructing a Stacked Long Short-Term Memory (SLSTM) neural network that contains “dropout”. We extracted features from protein sequences using a novel feature extraction scheme that combined Zernike Moments (ZMs) with Position Specific Weight Matrix (PSWM). The capability of the proposed approach was assessed on *S.erevisiae* and *Human* SIPs datasets. The result indicates that the approach based on deep learning can effectively resist data skew and achieve good accuracies of 95.69 and 97.88%, respectively. To demonstrate the progressiveness of deep learning, we compared the results of the SLSTM-based method and the celebrated Support Vector Machine (SVM) method and several other well-known methods on the same datasets.

**Conclusion:**

The results show that our method is overall superior to any of the other existing state-of-the-art techniques. As far as we know, this study first applies deep learning method to predict SIPs, and practical experimental results reveal its potential in SIPs identification.

## Background

As the embodiment of life activity, protein does not exist in isolation, but through interaction to complete most of the process in the cell. Protein-protein interaction (PPIs) has been the focus of the study of biological processes. SIPs are considered to be a unique protein interaction. SIPs have the same arrangement of amino acids. This leads to the formation of homodimer. Previous studies have proved that SIPs play a leading role in the discovering the laws of life and the evolution of protein interaction networks (PINs) [1]. It is important to understand whether proteins can interact with themselves, which helps clarify the function of proteins, insights into the regulation of protein function, and predicts or prevents disease. The homo-oligomerization have proven to play a significant role in the wide-ranging biological processes, for instance, immunological reaction, signal transduction, activation of enzyme, and regulation of gene expression [2–5]. It has been found that SIPs are a main aspect in regulating protein function by means of allosteric means. Many studies have shown that the diversity of proteins can be extended by SIPs without growing genome size. In addition, self-interaction helps to increase stability and prevent protein denaturation by reducing its surface area. SIPs have the potential to interact with many other proteins, hence, it occupies a significant position in cellular systems. SIPs have an ability to improve the stability of protein and avoid the denaturation of proteins and reduce its superficial area. An endless stream of experimental methods is used to detect protein self-interaction. However, these methods have certain drawbacks and limitations. It is urgent to develop an effective and reliable novel approach for predicting SIPs.

In recent years, some computational systems have been designed for predicting PPIs. Zaki et al. [6] projected a scheme for predicting SIPs that used only protein primary structure based on pairwise similarity theory. Zahiri J at el. [7] introduced an approach called PPIevo for predicting PPIs using a feature extraction algorithm. You et al. [8] gave a method called PCA-ELM that shows great ability in predicting PPIs. M. G. Shi et al. [9] shown a powerful method, which used correlation coefficient (CC) combined with support vector machine (SVM). This proposed method could be used in predicting PPIs, giving satisfactory results. These methods generally tend to use certain information about protein pairs, for instance, colocalization, coexpression and coevolution. Nevertheless, such feature is not applicable to deal with SIPs problems. Besides, the PPIs data sets adopted in above approaches do not cover SIPs. Hence, these computational-based methods not suitable for predicting SIPs. In the past research, Liu et al. [10] developed a prediction model to predict SIPs named as SLIPPER by mixing several typical known attributes. However, there is a major defect in this prediction model, which cannot deal with proteins that are not included in the current human interatomic. Given the limits of the above-mentioned approaches, it is needed to develop a more practical computational method for identifying SIPs.

In this study, a novel computational scheme based on deep learning named ZM-SLSTM is proposed for detecting SIPs from protein sequence. We firstly converted the SIPs sequence into Position Specific Weight Matrix (PSWM). Second, a novel feature extraction approach named as Zernike moments (ZMs) is adopted to generate feature vector from PSWM. Then, we build a Stacked Long Short-Term Memory (SLSTM) to predict SIPs. The proposed model was executed on *S.erevisiae* and *human* SIPs data sets. Satisfactory results are obtained with high accuracy of 95.69 and 97.88%, respectively. This method is also compared with other methods including Support Vector Machine (SVM), other (named as SLIPPER, CRS, SPAR DXECPPI, PPIevo and LocFuse). The results show that the ZM-SLSTM method perform better than any those methods. For all we know, our study is the first to adopt the deep-learning technology to predict SIPs, and experimental results show that our method can effectively resist data skew and improve the prediction performance relative to the existing technique.

## Method

### Datasets

We download 20,199 data of human sequences protein from the Uniprot database [11]. The PPIs data come from Various resource libraries including MatrixDB, BioGRID, DIP, IntAct and InnateDB [12–16]. In order to obtain the SIP data set, the PPI data that can interact with itself were collected. Accordingly, we obtained 2, 994 human SIPs sequences.

To collect datasets scientifically and efficiently, the human SIPs dataset is screened by the following steps [17]: (1) the protein sequence(>5000residues or < 50 residues) was removed from the whole human sequences protein; (2) For the construction of the positive data set, the selected SIPs must meet one of the following situations: (a) At least two mass experiments or one small scale experiment have shown that this protein sequence can interact with itself; (b) the protein must be homooligomer in UniProt; (c) the self-interaction of this protein have been reported by more than one publication; (3) For the sake of establish negative data set, all known SIPs were deleted from the whole human proteome.

As a result, 1441 human SIPs were selected to build positive data sets and 15,938 human protein that non-interacting were selected to build negative datasets. In addition, to better verify the usefulness of the designed scheme, we constructed the *S.erevisiae* SIPs dataset that cover 710 SIPs and 5511 non-SIPs by using above strategy.

### Position specific weight matrix

PSWM [18] was first adopted for detecting proteins of distantly related. The PSWM successfully applied in the field of biological information, including protein disulfide connectivity, protein structural classes, and subnuclear localization, DNA or RNA binding sites [19–23]. In the study, we used PSWM for predicting SIPs. A PSWM for a query protein is a Y×20 matrix *M* = {*m*_*ij*_: *i* =1 ⋯ Y *and* *j* = 1 ⋯ 20}, where the Y represents the size of the protein sequence and the number of columns of M matrix denotes 20 amino acids. In order to construct PSWM, a position frequency matrix is first created by calculating the presence of each nucleotide on each position. This frequency matrix can be represented as *p*(*u*, *k*), where *u* means position, *k* is the *k*_*th*_ nucleotide. The PSWM can be expressed as ${M}_{ij}={\sum}_{k=1}^{20}p\left(u,k\right)\times w\left(v,k\right)$, where *w*(*v*, *k*) is a matrix whose elements represent the mutation value between two different amino acids. Consequently, high scores represent highly conservative positions, and low points represent a weak conservative position.

In this paper, the PSWM of a protein sequences were generated by using Position specific iterated BLAST (PSI-BLAST) [24]. To get high and broad homologous information, we set three iterations and set the e-value to 0.001.

### Zernike moments

In this paper, the Zernike moments are introduced to extract meaningful information from protein sequence and generate feature vector [25–30]. We introduce the concept of the Zernike function to clearly define the moments of the Zernike. A set of complex polynomials are introduced by Zernike which form a complete orthogonal set within the unit circle. These polynomials are represented as *V*_*nm*_(*x*, *y*). These polynomials have the following form:

1 $$ {V}_{xy}\left(n,m\right)={V}_{xy}\left(\rho, \theta \right)={R}_{xy}\left(\rho \right){e}^{jy\theta}\mathrm{for}\ \rho \le 1 $$

where *x* is a positive integer greater than zero, *y* is integer, and satisfies |*y*| < *x*, where *x* - |*y*| is an even number. *ρ* is the length from (0, 0) to the pixel (*n*, *m*). *θ* represents included angle between vector *ρ* and *n* axis in counterclockwise direction. *R*_*xy*_(*ρ*)is

2 $$ {R}_{xy}\left(\rho \right)=\sum \limits_{s=0}^{\left(x-|y|/2\right)}{\left(-1\right)}^s\frac{\left(x-s\right)!}{s!\left(\frac{x+\left|y\right|}{2}-s\right)!\left(\frac{x+\left|y\right|}{2}-s\right)!}{\rho}^{x-2s} $$

From equation (2), we can find *R*_*x*, − *y*_(*ρ*) = *R*_*xy*_(*ρ*). These orthogonal polynomials are satisfying:

3 $$ \underset{0}{\overset{2\pi }{\int }}{\int}_0^1{V}_{xy}^{\ast}\left(\rho, \theta \right){V}_{pq}\left(\rho, \theta \right)\rho d\rho d\theta =\frac{\pi }{x+1}{\delta}_{xp}{\delta}_{yq}\kern0.75em $$

with

4 $$ {\delta}_{ab}=\left\{\begin{array}{c}1\ \\ {}0\end{array}\right.\kern2.25em \genfrac{}{}{0pt}{}{a=b}{otherwise} $$

The Zernike moments can be obtained by calculating (5)

5 $$ {Z}_{xy}=\frac{x+1}{\pi }{\sum}_{\left(\rho, \theta \right)\in unit\ circle}\sum f\left(\rho, \theta \right){V}_{nm}^{\ast}\left(\rho, \theta \right) $$

To calculate the ZMs of a protein sequence represented by a PSWM matrix, the origin is at the center of the matrix, and the points in the matrix are mapped inside the unit circle., i.e., *n*^2^ + *m*^2^ ≤ 1. The value falling outside the unit circle is not calculated [31–35]. Note that ${A}_{xy}^{\ast }={A}_{x,-y.}$

### Feature selection

**Fig. 1:**
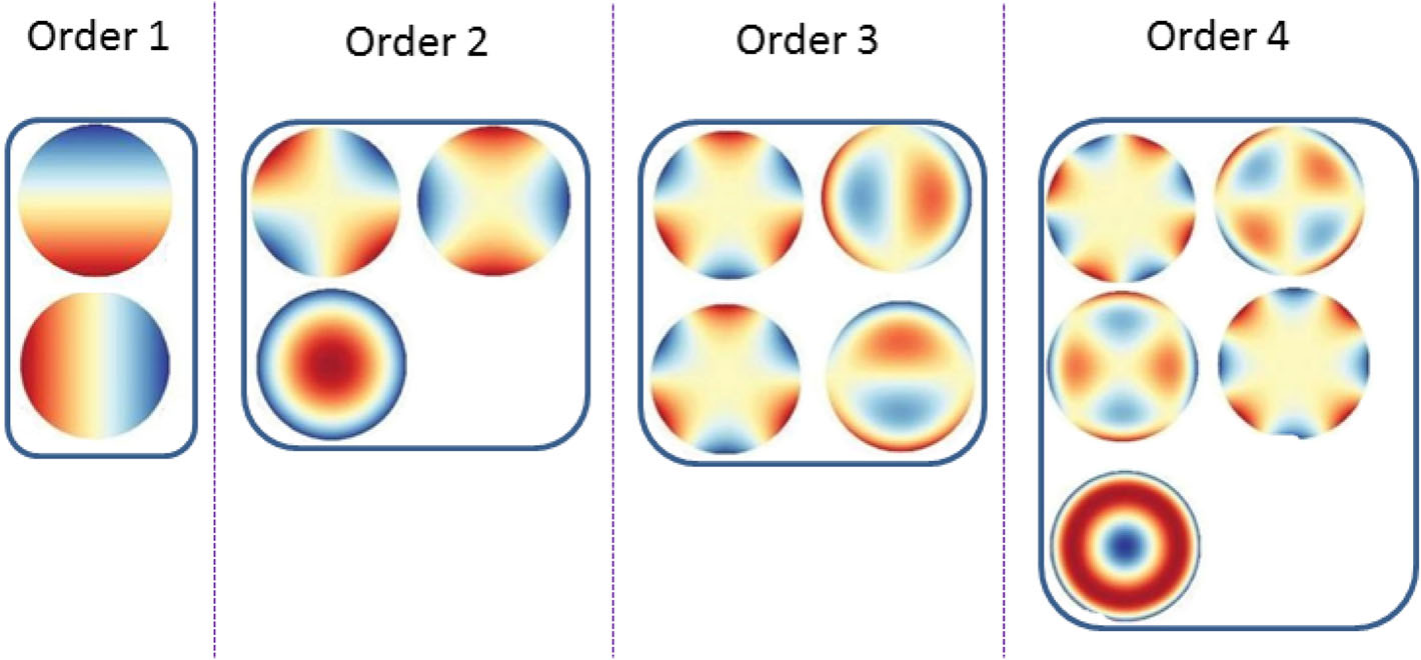
Plots of the magnitude of the Zernike moments with low order

To sum up, Zernike moments can extract some important information. When we use the Zernike moments, there is a problem that must be considered is how big *n*_*max*_should be set? The moments of lower order extract unsophisticated feature and the moments of higher order capture details feature. Figure 1 shows the magnitude plots of the Zernike moments with low order. Considering that we not only need enough information for more accurate classification, but also need to control the dimension of feature to reduce the computational cost. In this experiment, *x*_*max*_ is set to 30 [36–40]. This moment information constitutes the feature vectors of protein sequences

6 $$ \overrightarrow{F}={\left[\left|{A}_{11}\right|,\left|{A}_{22}\right|,\dots \dots, \left|{A}_{NM}\right|\right]}^T $$

where |*A*_*nm*_| represents the absolute value of Zernike moments. The zeroth order moments are not computed because they do not contain any valuable information and ZMs without considering *m* < 0, since they are inferred through ${A}_{n,-m}={A}_{nm}^{\ast }.$

Finally, in order to eliminate noise as much as possible and to reduce the computational complexity, the feature dimensional was reduced from 240 to 150 by means of principal component analysis (PCA) method [41].

### Long short-term memory

Long Short-Term Memory (LSTM), a special recurrent neural network, performs much better than standard recurrent neural networks in many tasks. Almost all exciting results based on recurrent neural networks are implemented by them. In this work, the deep LSTM net structure was first introduced to predict self-interaction protein.

**Fig. 2:**
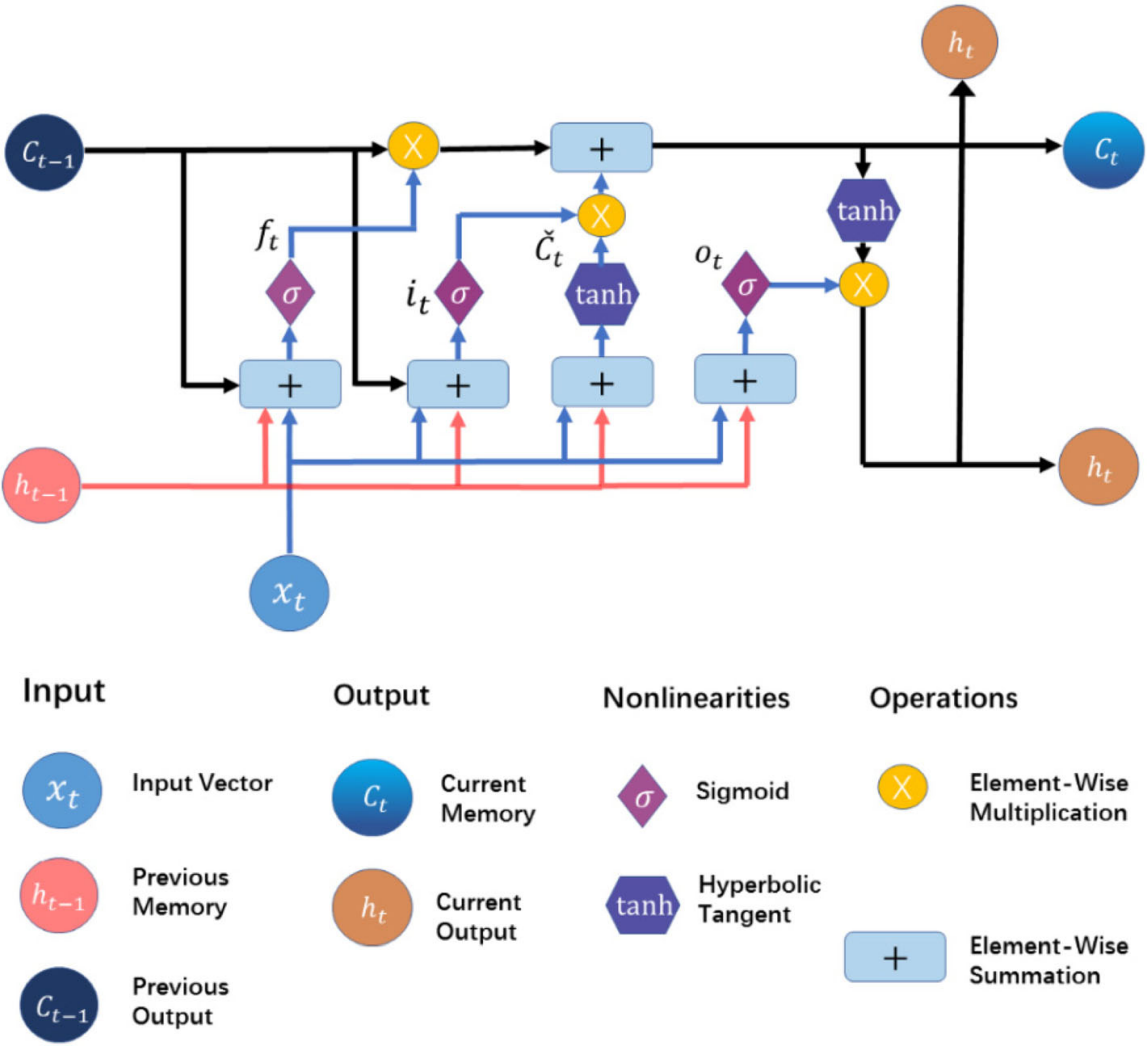
The structure of memory blocks in SLSTM networks

The main difference between LSTM network and other networks is its use of complex memory block instead of the neurons of general network. The memory block contains three multiplicative ‘gate’ units (the input, forget, and output gates.) along with some memory cells (one or more). The gate unit is used to control the information flow, and the memory cell is used to store the historical information [42–44]. The structure of the memory block is shown in the Fig. 2, to better understand the work of the gate unit, memory cells are not shown in the Fig. 2. The gate removes or restore information to the cell state by controlling the information flow. More specific, the input and output of the information flow are respectively handled by the input and output gates. The forget gate determines how much of the previous unit’s information is retained to the current unit. In addition, in order to enable memory blocks to store earlier information, we add a peephole to the block to connect the memory cell to the gate [45, 46].

The information flow passing through a memory block needs to do the following operations to complete the mapping from input *x* to output *h*:

7 $$ {i}_t= sigm\left({W}_i\bullet \left[{C}_{t-1},{x}_t,{h}_{t-1}\right]+{b}_i\right) $$

8 $$ {f}_t= sigm\left({W}_f\bullet \left[{C}_{t-1},{x}_t,{h}_{t-1}\right]+{b}_f\right) $$

9 $$ {o}_t= sigm\left({W}_o\bullet \left[{C}_t,{x}_t,{h}_{t-1}\right]+{b}_o\right) $$

10 $$ {\overset{\check{} }{C}}_t=\mathit{\tanh}\left({W}_C\bullet \left[{x}_t,{h}_{t-1}\right]+{b}_C\right) $$

11 $$ {C}_t={C}_{t-1}\ast {f}_t+{\overset{\check{} }{C}}_t\ast {i}_t $$

12 $$ {h}_t=\tanh \left({C}_t\right)\ast {o}_t+{\overset{\check{} }{C}}_t\ast {i}_t $$

Here, symbols related to the letter *C* represent cell activation vectors, the symbol *f, i*, *o*, and *C* are respectively the forget gate, input gate, output gate. The items related to *W* (*W*_*i*_, *W*_*f*_, *W*_*f*_), represent weight matrices, the items related to *b* (*b*_*i*_, *b*_*f*_, *b*_*o*_, *b*_*C*_) denote bias, *σ* is sigmoid function, ∗ is the element-wise product of the vectors.

### Stacked long short-term memory

**Fig. 3:**
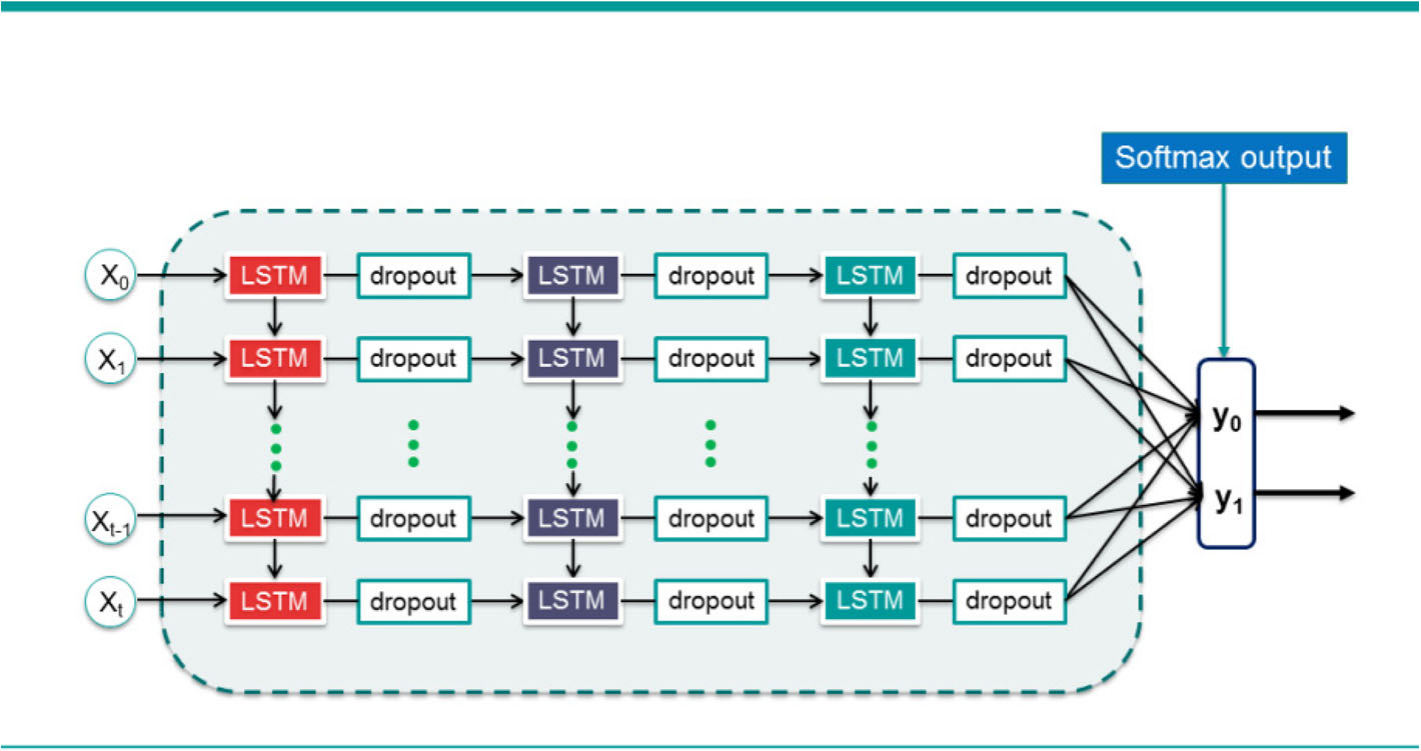
A Stacked Long Short-Term Memory network

A large number of theoretical and practical results support that the deep hierarchical network model can be more competent for complex tasks than shallow one. We construct the Stacked Long Short-Term Memory (SLSTM) net by stacking multiple LSTM hidden layers on top of each other, which contain one input layer, three LSTM hidden layers, one output layer. Figure 3 shows a SLSTM network. The number of neurons in the input layer is equal to the dimension of the input data. Each SLSTM hidden layer consist of 16 memory blocks. The number of neurons in the output layer equals the number of classes. Therefore, the number of neurons or memory blocks in each layer of the network are *200–16–16-16-2.* In output layer, the softmax function is used to generate probabilistic results.

### Prevent over fitting

**Fig. 4:**
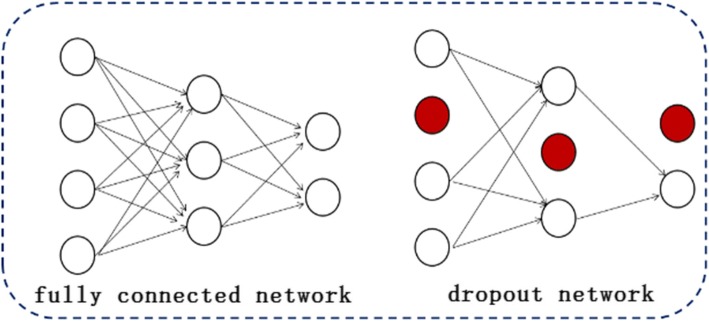
Network structure after using dropout

Overfitting problems exist in many prediction or classification models. Even the deep learning model with superior performance is no exception. A great deal of theoretical and practical work has proved that over-fitting can be reduced or avoided by adding “dropout” operation on neural net. “dropout” provides a way to approximate combine exponentially different neural network architectures [47]. More specific, “dropout” involves two important operations: 1) Dropout randomly discards hidden units and edges connected with them with a fixed probability in each training case; 2) In the test, dropout is responsible for integrating multiple neural networks generated during training. The first operation makes it possible to produce a different network almost every training case and these different networks share the same weights for the hidden units. The Fig. 4 describes a network model after using dropout. At test time, all hidden layer neurons are used without “dropout”, but the weight of the network is a reduced version of the trained weights. The proportion of weight reduction equals to the probability of the unit being retained [48]. By weight reduction, a large number of dropout networks can be merged into a single neural network and provide a similar performance to averaging over all networks [49].

## Results

### Performance evaluation

In order to evaluate the methods presented in this paper, we used a few commonly used indicators: The accuracy (ACC), true positive rate (TPR), positive predictive value (PPV), specificity (SPC), and Matthew’s Correlation Coefficient (MCC). The definition is given as follows:

13 $$ \mathrm{ACC}=\frac{TN+ TP}{TN+ FN+ TP+ FP} $$

14 $$ \mathrm{TPR}=\frac{TP}{FN+ TP} $$

15 $$ \mathrm{PPV}=\frac{TP}{TP+ FP} $$

16 $$ \mathrm{SPC}=\frac{TN}{TN+ FP} $$

17 $$ \mathrm{MCC}=\frac{\left( TP\times TN\right)+\left( FP\times FN\right)}{\sqrt{\left( TP+ FN\right)\times \left( TP+ FP\right)\times \left( TN+ FN\right)\times \left( TN+ FP\right)}} $$

where *TP* means those samples, have interacting, are predicted correctly, *FP* represents those samples, true non-interacting with each other, are judged to be interaction. *TN* represents those samples, true noninteracting with each other, are predicted correctly. *FN* represents those samples, true interacting with each other, are judged to be non-interacting. Furthermore, the Receiver operating characteristic (ROC) is portrayed to appraise the performance of a set of classification results and the AUC is computed as an important evaluation indicator [50, 51].

### Assessment of prediction

The proposed method is validated on two standard SIPs dataset. Each dataset is divide into three parts: The training set, accounted for 40 % of the total data; The verification set, accounts for 30 % of the total data; and the test set, accounts for 30 % of the total data. The training data sets are used to fit the weights of connections between memory block in the SLSTM network. The validation sets are used to fine tune model parameters and determine optimal performance models. Another function of the validation data set is to prevent overfitting by early stopping: when the errors on the validation data set begin to increase, the model stops training, because is a token of overfitting. The test data set is used for unbiased evaluation of the trained model. We train model only setting 200 epochs and using *Nadam* optimization method, that has more constraints on the learning rate, and also has a more direct impact on the gradient update.

**Fig. 5:**
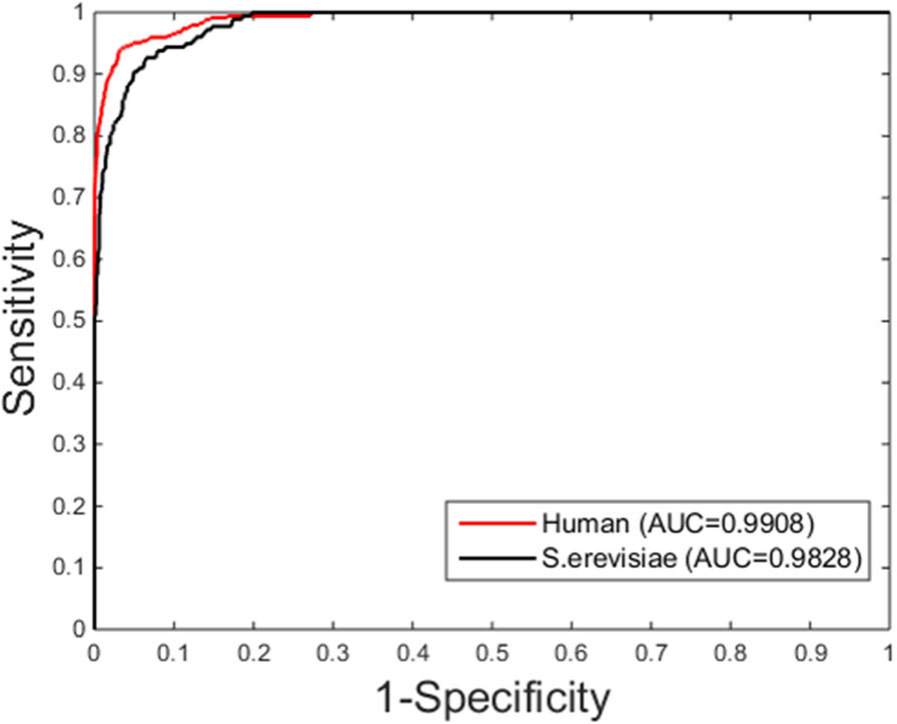
ROC curves achieved by the proposed approach

As Table 1 shows, the accuracy obtained by the ZMs-SLSTM is 95.69% for *S.erevisiae* and 97.88% for *Human* data sets. Beyond that, several other evaluation indicators also show the potential of our approach. More specifically, on *S.erevisiae,* the proposed method achieved TPR of 92.97%, SPC of 95.94%, PPV of 67.23%, MCC of 77.43% and AUC of 0.9828, respectively. For *Human* dataset with more samples, this method produces better results with TPR of 88.00%, SPC of 98.70%, PPV of 84.93%, MCC of 85.60% and AUC of 0.9908, respectively. The ROC curves achieved by the proposed ZMs-SLSTM method was exposed in Fig. 5.

**Table 1:** The results produced by the proposed method and the SVM-based method on PPIs datasets

Model	Data Sets	ACC (%)	TPR (%)	SPC (%)	PPV (%)	MCC (%)	AUC
SLSTM	*S.erevisiae*	95.69	92.97	95.94	67.23	77.43	0.9828
	*Human*	97.88	88.00	98.70	84.93	85.60	0.9908
SVM	*S.erevisiae*	93.06	57.22	97.68	76.25	64.59	0.9345
	*Human*	95.30	54.26	99.01	83.27	66.07	0.9261

### The performance of SVM-based approach

We verify the performance of our classifier by compare it with the SVM (Support Vector Machine) classifier representing the most advanced technologies. In this experience, we took the same feature extraction process in *S.erevisiae* and *Human* datasets, respectively. We used LIBSVM tools [52] to implement the classification of SVM. The SVM parameters of c and g are 0.5 *and* 0.6 by the grid search method.

Table 1 indicates, our ZMs-SLSTM method is significantly superior to SVM-based methods, particularly for predicting the true self-interacting protein pairs. Focus on *S.erevisiae* dataset, 95.69% ACC, 92.97% TPR, 77.43% MCC and 0.9828 AUC of the ZMs-SLSTM is much higher than the corresponding values for the SVM-predictor with 93.06% ACC, 57.22%TPR, 64.59% MCC and 0.9345. AUC. Similar situations also appear on the *Human* data set, the performance of the ZMs-SLSTM method has been found to be better with 97.88% ACC, 88.00% TPR, 98.70% SPC, 84.93% PPV, 85.60% MCC and 0.9908 AUC versus 95.30% ACC, 54.26% TPR, 99.01% SPC, 83.27% PPV, 66.07% MCC and 0.9261 AUC, respectively. In particular, higher TPR (92.97% on *S.erevisiae* dataset and 88.00% on *Human* dataset) indicates our method can give more accurate results than SVM-based approach (57.22% on *S.erevisiae* dataset and 54.26% on *Human* dataset) in predicting true SIPs.

### Comparison with other methods

To further evaluate our proposed approach, we also compared it with six existing methods (SLIPPER, CRS, SPAR, DXECPPI, PPIevo and LocFuse). Table 2 presents the results of several methods on *S.erevisiae* and *Human* data sets. From Table 2, compared with other methods, our method significantly improves the overall performance of the SIPs prediction. In addition, SLIPPER contains some restrictions. Second, it integrates a large amount of known knowledge, such as GO terms, PINs, drug targets, and enzymes. In particular, the degree of protein in the PIN makes a significant contribution to SIP predictions. However, for unknown or artificial proteins in actual applications, all information is difficult to access directly. Therefore, as long as the protein sequence is known, our method is necessary for improved SIP prediction. DXECPPI is a PPI predictor, because the traditional PPI predictor uses correlation information between two proteins, such as co-expression, co-evolution and co-localization, and cannot be effectively used for SIP prediction. Therefore, our method can be used as a necessary supplement for PPI prediction. For *S.erevisiae* data set, the method presented by this paper achieves the best accuracy of 95.69%, which is much higher than that of other methods. More obvious improvements are reflected in TPR, MCC, and AUC. Observe the results on the *S.erevisiae* data set, 92% TPR achieved by the ZMs-SLSTM approach is more than three triple that of the DXECPPI method, and 77.43% MCC achieved by the ZMs-SLSTM approach is more than four triple that of the PPIevo method. 0.9828 AUC achieved by the ZMs-SLSTM approach is 37% higher than the average of other methods. High TPR shows that our method has little error rate in identifying self-interacting proteins. The high MCC and AUC show that our model is robust, practical, and can effectively resist data skew. The SIP prediction for *Human* dataset (Table 2) have also been greatly improved by using our approach. 97.88% ACC, 85.60% MCC and 0.9908 AUC of the ZMs-SLSTM is are way above the corresponding values for the other method. In addition, compared the results of SVM-based method (Table 1) and six existing methods (SLIPPER, CRS, SPAR, DXECPPI, PPIevo and LocFuse), it can be found that our method is still overall superior to the six existing predictors. This shows that the proposed feature extraction strategy proposed in this paper is efficient, useful and plays an important role in the SIPs prediction model. The results of this study illustration that the ZMs-SLSTM approach is capable of effectively improving the prediction performance of SIPs.

**Table 2:** Performance comparison of seven approaches on both the *S.erevisiae* and *Human* datasets

Methods	*S.erevisiae*					*Human*				
	ACC (%)	SPC (%)	TPR (%)	MCC (%)	AUC	ACC (%)	SPC (%)	TPR (%)	MCC (%)	AUC
SLIPPER	71.90	72.18	69.72	28.42	0.7723	91.10	95.06	47.26	41.97	0.8723
DXECPPI	87.46	94.93	29.44	28.25	0.6934	30.90	25.83	87.08	8.25	0.5806
PPIevo	66.28	87.46	60.14	18.01	0.6728	78.04	25.82	87.83	20.82	0.7329
LocFuse	66.66	68.10	55.49	15.77	0.7087	80.66	80.50	50.83	20.26	0.7087
CRS	72.69	74.37	59.58	23.68	0.7115	91.54	96.72	34.17	36.33	0.8196
SPAR	76.96	80.02	53.24	24.84	0.7455	92.09	97.40	33.33	38.36	0.8229
ZM-SLSTM	95.69	95.94	92.97	77.43	0.9828	97.88	98.70	88.00	85.60	0.9908

## Discussion

This method can produce good results mainly due to: effective feature extraction strategy and reliable classifiers.

The protein feature extraction scheme consisting of PSWM and ZMs effectively captures the evolutionary information of protein and produces the most characteristic features that improve the ability of the classifier to distinguish unknown samples during the testing phase. The robust and efficient SLSTM deep neural network also make a great contribution to accuracy improvement that provide stronger classification performance than traditional machine learning method in interaction pattern recognition. The performance improvement brought by SLSTM comes mainly from the following reasons: 1) Compared with the traditional machine learning methods, the hierarchical structure of deep learning algorithms can process more complex data, and automatically learn abstract and more useful features. 2) Two mechanisms to prevent overfitting, dropout and early stopping, make the prediction model trained more reliable, robust and excellent. 3) In the testing phase, we merged all dropout networks generated by the training processes, which led to a better result. 4) The SLSTM network uses memory blocks instead of simple neurons, which allows the network to learn more knowledge about self-interacting proteins during training.

## Conclusion

In recent years, the rise of deep learning technology has constantly affected the development of various fields. However, the ability of deep learning techniques in predicting self-interacting proteins has not been witnessed. In this work, a SLSTM neural network was constructed as a deep learning model to predict SIPs only using protein sequences. The method is applied to two standard data sets and the results show it is reliable, stable and accurate for predicting SIPs. The contribution of the proposed approach comes mainly from three technologies: SLSTM network, ZMs feature extractor, PSWM. Specifically, each protein sequence was converted into PSWM by using PSI-BLAST. The ZMs then is adopted to catch the valuable information from PSWM and form feature vectors that as input of classifier. Finally, the SLSTM deep network is used to predict SIPs. For further measuring the performance of the ZMs-SLSTM method, ZMs-SVM and other six methods were implemented on *S.erevisiae* and *Huamn* data sets for comparing with the proposed approach. The results from these experiments indicate that the SIPs detection capability of the proposed scheme is overall ahead of that of the earlier methods and SVM-based approach. The performance improvement caused by this method is mainly dependent on the use of an excellently deep learning model and a fresh and high-performance feature extraction scheme. To the best of our knowledge, this study is the first to build a deep learning model for SIP prediction using protein sequence, and the results demonstrate our method is strong and practical.

## Abbreviations

ACC: Accuracy
MCC: Matthew’s correlation coefficient
PINs: Protein interaction networks
PPIs: Protein-protein interaction
PPV: Positive predictive value
PSWM: Position specific weight matrix
SIPs: Self-interacting proteins
SLSTM: Stacked long short-term memory
SPC: specificity
SVM: Support vector machine
TPR: True positive rate
ZMs: Zernike moments
